# Virtual and Actual Humanoid Robot Control with Four-Class Motor-Imagery-Based Optical Brain-Computer Interface

**DOI:** 10.1155/2017/1463512

**Published:** 2017-07-18

**Authors:** Alyssa M. Batula, Youngmoo E. Kim, Hasan Ayaz

**Affiliations:** ^1^Department of Electrical and Computer Engineering, Drexel University, 3141 Chestnut Street, Philadelphia, PA 19104, USA; ^2^School of Biomedical Engineering, Science and Health Systems, Drexel University, 3141 Chestnut Street, Philadelphia, PA 19104, USA; ^3^Department of Family and Community Health, University of Pennsylvania, 3737 Market Street, Philadelphia, PA 19104, USA; ^4^Division of General Pediatrics, Children's Hospital of Philadelphia, 3401 Civic Center Blvd, Philadelphia, PA 19104, USA

## Abstract

Motor-imagery tasks are a popular input method for controlling brain-computer interfaces (BCIs), partially due to their similarities to naturally produced motor signals. The use of functional near-infrared spectroscopy (fNIRS) in BCIs is still emerging and has shown potential as a supplement or replacement for electroencephalography. However, studies often use only two or three motor-imagery tasks, limiting the number of available commands. In this work, we present the results of the first four-class motor-imagery-based online fNIRS-BCI for robot control. Thirteen participants utilized upper- and lower-limb motor-imagery tasks (left hand, right hand, left foot, and right foot) that were mapped to four high-level commands (turn left, turn right, move forward, and move backward) to control the navigation of a simulated or real robot. A significant improvement in classification accuracy was found between the virtual-robot-based BCI (control of a virtual robot) and the physical-robot BCI (control of the DARwIn-OP humanoid robot). Differences were also found in the oxygenated hemoglobin activation patterns of the four tasks between the first and second BCI. These results corroborate previous findings that motor imagery can be improved with feedback and imply that a four-class motor-imagery-based fNIRS-BCI could be feasible with sufficient subject training.

## 1. Introduction

The ability to direct a robot using only human thoughts could provide a powerful mechanism for human-robot interaction with a wide range of potential applications, from medical to search-and-rescue to industrial manufacturing. As robots become more integrated into our everyday lives, from robotic vacuums to self-driving cars, it will also become more important for humans to be able to reliably communicate with and control them. Current robots are difficult to control, often requiring a large degree of autonomy (which is still an area of active research) or a complex series of commands entered through button presses or a computer terminal. Using thoughts to direct a robot's actions via a brain-computer interface (BCI) could provide a more intuitive way to issue instructions to a robot. This could augment current efforts to develop semiautonomous robots capable of working in environments unsafe for humans, which was the focus of a recent DARPA robotics challenge [1]. A brain-controlled robot could also be a valuable assistive tool for restoring communication or movement in patients with a neuromuscular injury or disease [2].

The ideal, field-deployable BCI system should be noninvasive, safe, intuitive, and practical to use. Many previous studies have focused on electroencephalography (EEG) and, to a lesser extent, functional magnetic resonance imaging (fMRI). Using these traditional neuroimaging tools, various proof-of-concept BCIs have been built to control the navigation of humanoid (i.e., human-like) robots [3–9], wheeled robots [10–12], flying robots [13, 14], robotic wheelchairs [15], and assistive exoskeletons [16]. More recently functional near-infrared spectroscopy (fNIRS) has emerged as a good candidate for next generation BCIs, as fNIRS measures the hemodynamic response similar to fMRI [17, 18] but with miniaturized sensors that can be used in field settings and even outdoors [19, 20]. It also provides a balanced trade-off between temporal and spatial resolution, compared to fMRI and EEG, that sets it apart and presents unique opportunities for investigating new approaches, mental tasks, information content, and signal processing for the development of new BCIs [21]. Several fNIRS-based BCI systems have already been investigated for use in robot control [22–27].

Motor imagery, or the act of imagining moving the body while keeping the muscles still, has been a popular choice for use in BCI studies [3, 11, 13, 22, 23, 28–37]. It is a naturalistic task, highly related to actual movements, which could make it a good choice for a BCI input. While motor-execution tasks produce activation levels that are easier to detect, motor imagery is often preferred as issues with possible proprioceptive feedback can be avoided [38]. EEG BCIs have shown success with up to four classes, typically right hand, left hand, feet, and tongue [11, 28, 29]. Other studies have shown potential for EEG to detect difference between right and left foot or leg motor imagery [39, 40] and even individual fingers [41]. Studies have also used fNIRS to detect motor-imagery tasks, with many focusing on a single hand versus resting state [30], left hand versus right hand [31, 32], or three motor-imagery tasks and rest [33]. Shin and Jeong used fNIRS to detect left and right leg movement tasks in a four-class BCI [42], and in prior studies we presented preliminary offline classification results using left and right foot tasks separately in a four-class motor-imagery-based fNIRS-BCI [22, 23]. fNIRS has also been used to examine differences in motor imagery due to force of hand clenching or speed of tapping [34].

Many factors can affect the quality of recorded motor-imagery data. Kinesthetic motor imagery (i.e., imagining the feeling of the movement) has shown higher activation levels in the motor cortex than visual motor imagery (i.e., visualizing the movement) [43, 44]. Additionally, individual participants have varying levels of motor-imagery skill, which also affects the quality of the BCI [45–47]. In some participants, the use of feedback during motor-imagery training can increase the brain activation levels produced during motor imagery [48, 49].

Incorporating robot control into a BCI provides visual feedback and can increase subject motivation. Improved motivation and feedback, both visual and auditory, have demonstrated promise for reducing subject training time and improving BCI accuracy [50, 51]. The realism of feedback provided by a BCI may also have an effect on subject performance during motor imagery. For example, Alimardani et al. found a difference in subject performance in a follow-up session after receiving feedback from viewing a robotic gripper versus a lifelike android arm [52].

In this study, we report the first online results of a four-class motor-imagery-based fNIRS-BCI used to control both a virtual and physical robot. The four tasks used were imagined movement of upper and lower limbs: the left hand, left foot, right foot, and right hand. To the best of our knowledge, this is the first online four-class motor-imagery-based fNIRS-BCI, as well as the first online fNIRS-BCI to use left and right foot as separate tasks. We also examine the differences in oxygenated hemoglobin (HbO) activation between the virtual and physical-robot BCIs in an offline analysis.

## 2. Materials and Methods

Participants attended two training sessions, to collect data to train an online classifier for the BCI, followed by a third session in which they used the BCI to control the navigation of both a virtual and actual robot. This section outlines the methods used for data collection, the design of the BCI, and offline analysis of the collected data following the completion of the BCI experiment.

### 2.1. Participants

Thirteen healthy participants volunteered to take part in this experiment. Subjects were aged 18–35, right-handed, English-speaking, and with vision correctable to 20/20. No subjects reported any physical or neurological disorders or were on medication. The experiment was approved by the Drexel University Institutional Review Board, and participants were informed of the experimental procedure and provided written consent prior to participating.

### 2.2. Data Acquisition

Data were recorded using fNIRS as described in our previous study [53]. fNIRS is a noninvasive, relatively low-cost, portable, and potentially wireless optical brain imaging technique [19]. Near-infrared light is used to measure changes in HbO and HbR (deoxygenated hemoglobin) levels due to the rapid delivery of oxygenated blood to active cortical areas through neurovascular coupling, known as the hemodynamic response [54].

**Figure 1:**
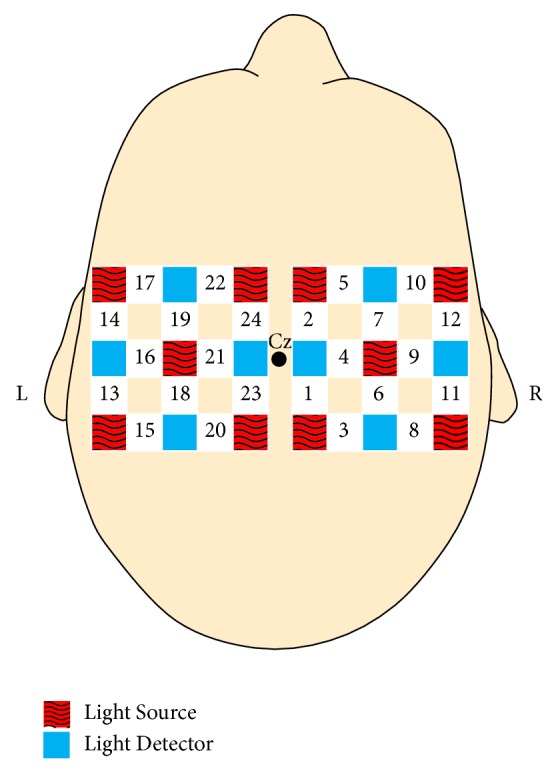
fNIRS sensor layout of light sources (red squares) and detectors (blue squares). Adjacent sources and detectors are 3 cm apart and create 24 optodes (numbered 1–24).

Participants sat in a desk chair facing a computer monitor. They were instructed to sit with their feet flat on the floor and their hands in their lap or on chair arm rests with palms facing upwards. Twenty-four optodes (measurement locations) over the primary and supplementary motor cortices were recorded using a Hitachi ETG-4000 optical topography system, as shown in Figure 1. Each location recorded HbO and HbR levels at a 10 Hz sampling rate.

### 2.3. Experiment Protocol

Motor-imagery and motor-execution data were recorded in three one-hour-long sessions on three separate days. The first two sessions were training days, used to collect initial data to train a classifier, and the third day used this classifier in a BCI to navigate both a virtual and physical robot to the goal location in a series of rooms. The two robots are described below in Section 2.3.3 Robot Control. The training session protocol included five tasks: a “rest” task and tapping of the right hand, left hand, right foot, and left foot. This protocol expands on a preliminary study reported previously [22, 23]. Data collection for the two training days has been described previously [53].

#### 2.3.1. Tasks

Subjects performed all five tasks during the two training days (rest, along with the (actual or imagined) tapping of the right hand, left hand, right foot, and left foot). During the third session, only the four motor-imagery tasks were used to control the BCI.

Participants were instructed to self-pace their real or imagined movements at once per second for the duration of the trial. The hand-tapping task was curling and uncurling their fingers towards their palm as if squeezing an imaginary ball, while the foot-tapping task involved raising and lowering the toes while keeping the heel on the floor. While resting, subjects were instructed to relax their mind and refrain from moving. During motor-imagery tasks, subjects were instructed to refrain from moving and use kinesthetic imagery (i.e., imagine the feelings and sensations of the movement).

**Figure 2:**
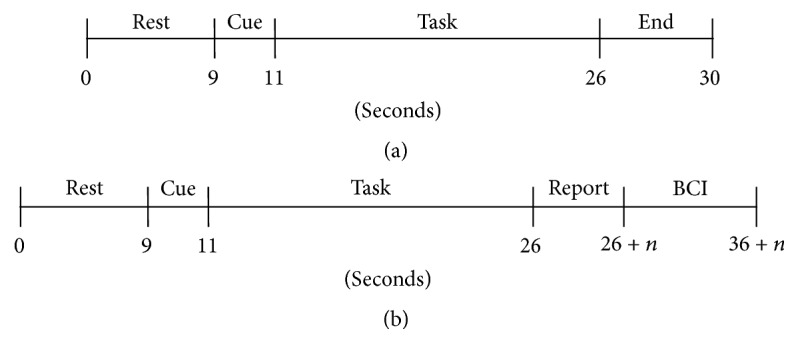
Trial timing diagrams for training sessions (a) and robot-control session (b).

Each trial consisted of 9 seconds of rest, a 2-second cue indicating the type of upcoming task, and a 15-second task period. During the two training sessions the cue text indicated a specific task (e.g., “Left Foot”), while, during the robot-control task, it read “Free Choice,” indicating the subject should choose the task corresponding to the desired action of the robot. Trials during the training days ended with a 4-second display indicating that the task period had ended. During the robot-control session, the task was followed by a reporting period so that the subject could indicate which task they had performed. The BCI then predicted which task the user had performed and sent the corresponding command to the robot, which took the corresponding action. The timings for training and robot-control days are shown in Figure 2.

#### 2.3.2. Session Organization

**Figure 3:**
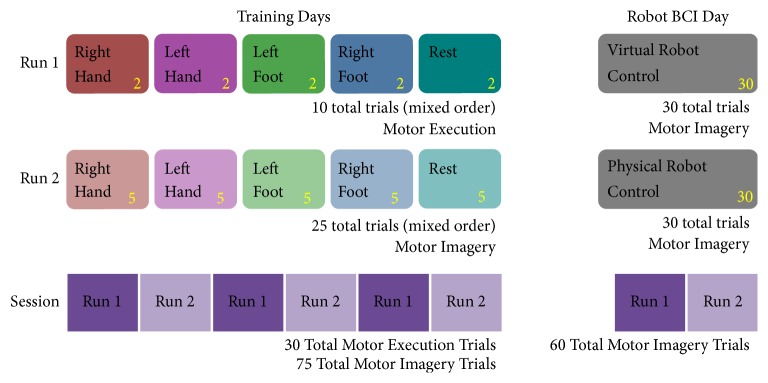
Trial organization protocol for the two training days and single robot-control day of the experiment.

In total, 60 motor-execution and 150 motor-imagery trials were collected during the training days, and an additional 60 subject-selected motor-imagery trials were recorded during the robot-control portion. The two training days were split into two runs, one for motor execution and one for motor imagery, which were repeated three times as shown in Figure 3. The protocol alternated between a run of 10 motor-execution trials and a run of 25 motor-imagery trials in order to reduce subject fatigue and improve their ability to perform motor imagery [55]. Each run had an equal number of the five tasks (rest and motor execution or motor imagery of the right hand, left hand, right foot, and left foot) in a randomized order. The third day (robot control) had two runs of 30 motor-imagery tasks, chosen by the user, which were used to control the BCI. The rest and motor-execution tasks were collected for offline analysis and were not used in the online BCI.

#### 2.3.3. Robot Control

**Figure 4:**
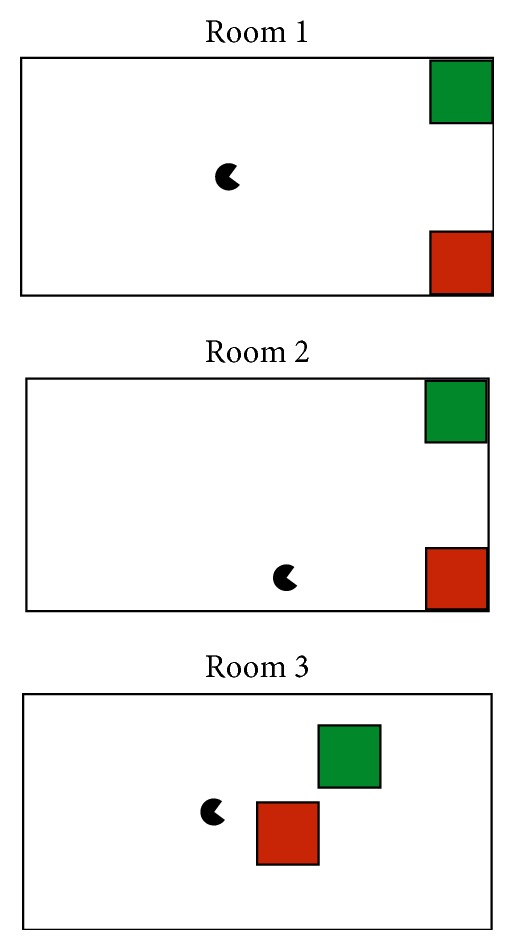
The three room layouts used during robot control.

The robot-control session had two parts, beginning with control of a virtual robot using the MazeSuite program (http://www.mazesuite.com) [56, 57] and followed by control of the DARwIn-OP (Dynamic Anthropomorphic Robot with Intelligence-Open Platform) robot [58]. The objective in both scenarios was to use the BCI to navigate through a series of three room designs (shown in Figure 4), in which there was a single goal location (a green cube) and an obstacle (a red cube). A room was successfully completed if the user navigated the robot to the green cube, and it failed if the robot touched the red cube. After completion or failure of a room, the subject would advance to the next room. The sequence was designed such that the robot started closer to the obstacle in each successive room to increase the difficulty as the subject progressed. The run ended if the subject completed (or failed) all three rooms or reached the maximum of 30 trials. Each room could be completed in 5 or fewer movements, assuming perfect accuracy from the BCI.

**Figure 5:**
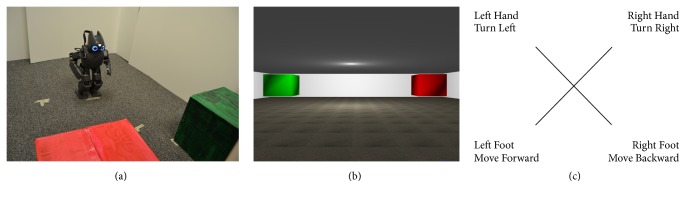
The DARwIn-OP robot standing at the starting location of the first room (a), the first-person display of the virtual room (b), and the experiment display showing the mappings between motor-imagery tasks and robot commands (c).

To control the BCI, subjects selected a motor-imagery task corresponding to the desired action of the (virtual or physical) robot. The task-to-command mappings were as follows: left foot/walk forward, left hand/turn left 90°, right hand/turn right 90°, and right foot/walk backward. These four tasks were chosen to emulate a common arrow-pad setup, so that each action had a corresponding opposite action that could undo a movement. During BCI control, the original experiment display showed a reminder of the mapping between the motor-imagery tasks and the robot commands. A second monitor to the left of the experiment display showed a first-person view of the experiment room for either the virtual or physical robot. The experiment setup and example display screens are shown in Figure 5.

The virtual robot was controlled using the built-in control functions of the MazeSuite program [56, 57]. The virtual environment and movements of the virtual robot were designed to replicate as closely as possible the physical room and movements of the DARwIn-OP, allowing the participants to acquaint themselves with the new robot-control paradigm before adding the complexities inherent in using a real robot. The virtual robot could make perfect 90° turns in place, and the forward and backward distance was adjusted to match the relative distance traveled by the DARwIn-OP robot as closely as possible. The goal and obstacle were shown as floating green and red cubes, respectively, that would trigger a success or failure state on contact with the virtual robot.

During the second run, the user controlled the DARwIn-OP in an enclosed area with a green box and red box marking the location of the goal and obstacle, respectively. Success or failure was determined by an experimenter watching the robot during the experiment. The DARwIn-OP is a small humanoid robot that stands 0.455 m tall, has 20 degrees of freedom, and walks on two legs in a similar manner to humans [58]. The robot received high-level commands from the primary experiment computer using TCP/IP over a wireless connection. Control of the DARwIn-OP was handled via a custom-built C++ class that called the robot's built-in standing and walking functions using prespecified parameters to control the movements at a high level. This class was then wrapped in a Python class for ease of communication with the experiment computer. The head position was lowered from the standard walking pose, in order to give a better view of the goal and obstacle. In order to turn as closely to 90° in place as possible, the robot used a step size of zero for approximately 3 seconds with a step angle of approximately 25° or −25°. When moving forward or backward, the DARwIn-OP used a step size of approximately 1 cm for 2 or 3 seconds, respectively. The exact values were empirically chosen for this particular robot.

### 2.4. Data Analysis

In addition to the evaluation of the classifier performance during the online BCI, a secondary offline analysis of the data was performed to further compare the two robot BCIs.

#### 2.4.1. Online Processing

Motor-imagery data from the two training days were used to train a subject-specific classifier to control the BCI during the third day. The rest and motor-execution trials were excluded from the training set, as the BCI only used the four motor-imagery tasks. All data recordings from the training days for HbO, HbR, and HbT (total hemoglobin) were filtered using a 20th-order FIR filter with a 0.1 Hz cutoff. Artifacts and optodes with poor signal quality were noted and removed by the researcher. One subject was excluded from the online results due to insufficient data quality.

In addition to using only the low-pass filter, a variety of preprocessing methods were evaluated: correlation-based signal improvement (CBSI), common average referencing (CAR), task-related component analysis (TRCA), or both CAR and TRCA. CBSI uses the typically strong negative correlation between HbO and HbR to reduce head motion noise [59]. CAR is a simple method, commonly used in EEG, in which the average value of all optodes at each time point is used as a common reference (i.e., that value is subtracted from each optode at that time point). This enhances changes in small sets of optodes while removing global spatial trends from the data. TRCA creates signal components from a weighted sum of the recorded data signals [60]. It attempts to find components that maximize the covariance between instances of the same task while minimizing the covariance between instances of different tasks.

**Figure 6:**
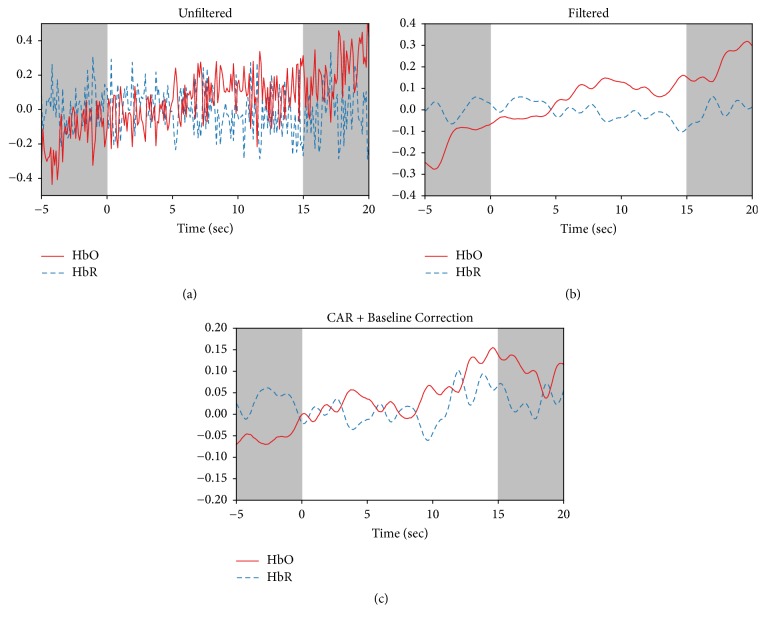
Example of data analysis for a representative trial. Data from a single optode showing the original HbO and HbR signals (a), the data after filtering (b), and after applying CAR and baseline correction (c). Resting periods before and after the task are shown by gray boxes.

Individual task periods were extracted and baseline corrected, using the first 2 seconds of each task as the baseline level.  Figure 6 shows an example of how preprocessing methods affect the recorded HbO and HbR for a single optode during one task period. Comparing Figures 6(a) and 6(b) shows how filtering removes a significant quantity of high-frequency noise from the signal.  Figure 6(c) shows the change in the signal after applying CAR and baseline correction.

Four different types of features were calculated individually on each optode for HbO, HbR, and HbT. The features used were as follows: mean (average value of the last 10 seconds of the task), median (median of the last 10 seconds of the task), max (maximum value of the last 10 seconds of the task), and slope (slope of the line of best fit of the first 7 seconds of the task). Datasets were created using features calculated on HbO, HbT, or both HbO and HbR. Each feature set was reduced to between 4 and 8 features using recursive feature elimination. If both HbO and HbR were used, the specified number of features was selected for each chromophore. This resulted in 300 possible datasets (5 preprocessing methods, 3 chromophore combinations, 4 types of features, and 5 levels of feature reduction). Features in each dataset were normalized to have zero mean and unit variance.

**Figure 7:**
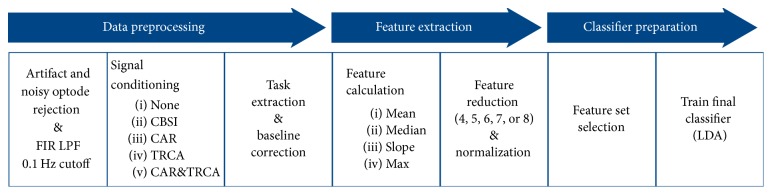
Flow chart outlining the creation of the online classifier.

Prior to the BCI session, a linear discriminant analysis (LDA) classifier was trained on the data from the two training days, following the flow chart shown in Figure 7 [61]. LDA is one of the simplest classification methods commonly used in BCIs [38], requiring no parameter tuning, which reduces the number of possible choices when selecting a classifier. LDA was implemented using the Scikit-learn toolkit [62].

To select an online classifier, an LDA classifier was trained on one training day (60 motor-imagery trials) and tested on the other for each of the 300 feature sets. This was repeated with the two days reversed, and the feature set with the highest average accuracy was selected. The classifier was then retrained on both training days (120 motor-imagery trials) using the selected feature set and was used as the online classifier for both robot-control BCIs.

Results are reported as accuracy (average number of correct classifications), precision (positive prediction value), recall (sensitivity or true positive rate), *F*-score (the balance between precision and recall), and the area under the ROC curve (AUC). The *F*-score is calculated as *F*-Score = 2 × (precision × recall)/(precision + recall).

#### 2.4.2. Offline Processing

For the offline analysis, an automatic data-quality analysis was used on the BCI session data to determine which optodes and trials should be removed due to poor quality. This was done separately for the virtual and DARwIn-OP runs using a modified version of the method described by Takizawa et al. for fNIRS data [63]. Any optodes with a very high (near maximum) digital or analog gain were removed, as these were likely contaminated by noise. Areas with a standard deviation of 0 in a 2-second window of the raw light-intensity data were considered to have been saturated, and artifacts were determined to be areas with a change of 0.15 [mM] during a 2-second period on HbO and HbR data after application of the low-pass filter. Optodes that had at least 20 (of the original 30) artifact- and saturation-free trials were kept, with the remaining optodes being removed. Then, any trials with artifacts or saturated areas in any remaining good optodes were removed. An additional 5 subjects were excluded from the offline analysis due to insufficient data quality.

CAR was used for all offline analysis, followed by task data extraction and baseline correction as in the online analysis. Offline analysis examined the average HbO activation levels during the first and last second of each trial. Statistical analysis was done using linear mixed models, with multiple tests being corrected using false discovery rate (FDR).

## 3. Results

Offline analysis found that optode (24 levels), the interaction of optode and task (4 levels: right hand, left hand, right foot, and left foot), and the interaction of optode and robot type (2 levels: virtual and DARwIn-OP) had a significant effect on the average HbO activation during the last second of each trial. A post hoc analysis run individually for each optode found no significant effect for task, robot type, or task*∗*robot type interaction. *F*-values and *p* values for the main effects are shown in Table 1, with the post hoc analysis available in Table S1 in Supplementary Material available online at https://doi.org/10.1155/2017/1463512.

A second post hoc analysis, run individually for each optode under each task condition separately, showed that robot type had a significant effect on at least one optode under each task condition (*p* < 0.05, FDR corrected). The effect was found for two optodes (14 and 16) for the left hand task, one optode (14) for left foot, 6 optodes (4, 9, 16, 18, 20, and 23) for right foot, and one optode (6) for right hand. The full table of *p* values is available in Table S2 in Supplementary Material.

A comparison of topographic HbO activation levels demonstrated differences between individual tasks as well as the two BCIs. Left hand showed a much more contralateral activation pattern with the DARwIn-OP robot, with two optodes on the ipsilateral side showing a significant decrease in HbO levels between the first and last second of the task, whereas, during control of the virtual robot, it had a more ipsilateral activation pattern and no optodes with statistically significant changes in activation over the course of the task. Right hand, however, became strongly ipsilateral, with one ipsilateral optode showing significant activation, during the DARwIn-OP BCI.

Right foot activation became more contralateral, with stronger activation being closer to C_z_ on the contralateral side and a significant decrease in activation on the ipsilateral side. Left foot changed from a centralized bilateral activation near C_z_ when controlling the virtual robot to a more diffuse and ipsilateral activation pattern during DARwIn-OP control. It did, however, show an optode with significant decrease in HbO activation on the ipsilateral side during DARwIn-OP control.

**Figure 8:**
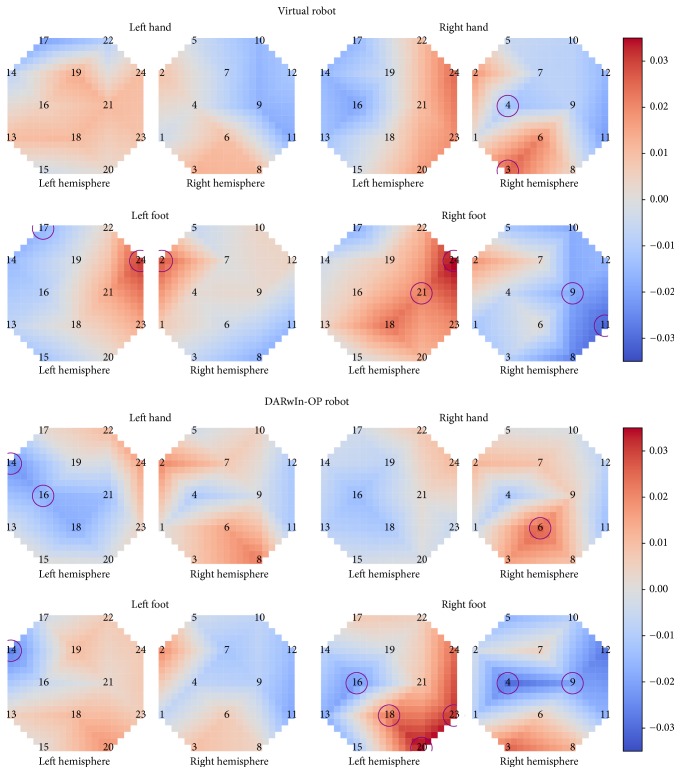
Average HbO activation for each task during virtual and DARwIn-OP robot BCIs. Optodes with significant differences in HbO activation levels between the first and last second of the task are circled (*p* < 0.05, FDR corrected).

Topographic plots of the average HbO activation during the last second of each task across all subjects are shown in Figure 8. Optodes showing a significant difference in average HbO level between the first and last second of the task are circled (*p* < 0.05, FDR corrected).

While controlling the online four-class BCI, participants achieved an average accuracy of 27.12% for the entire session. Five participants (S1, S5, S7, S8, and S11) achieved an accuracy of 30% or higher, reaching 36.67% accuracy (S8). The online accuracy, precision, recall, *F*-Score, and AUC for each subject are detailed in Table 2.

There was a significant increase in classification accuracy during DARwIn-OP control as compared to virtual robot control (one-sided paired* t*-test, *t*(11) = 2.077, *p* = 0.031), with the average accuracy increasing by 5.21 +/− 2.51% (mean +/− standard error). All but one subject achieved the same or better performance in the second run while controlling the DARwIn-OP compared to during the first run with the virtual robot, and two subjects achieved 40% accuracy. The online accuracy, precision, recall, *F*-Score, and AUC for each subject for each BCI individually are detailed in Table 3. One subject (S5) did not use the left hand task during the virtual robot run, and therefore no AUC value is listed.

This improvement in performance appears to be reflected in the number of goals reached by the participants. While controlling the virtual robot, subject S11 was the only participant to run into an obstacle, and they were also the only participant to reach a goal. During control of the DARwIn-OP robot, two subjects (S2 and S5) reached two of the goals, and two others (S1 and S11) reached a single goal. Two subjects (S1 and S7) collided with an obstacle while navigating the DARwIn-OP.

**Figure 9:**
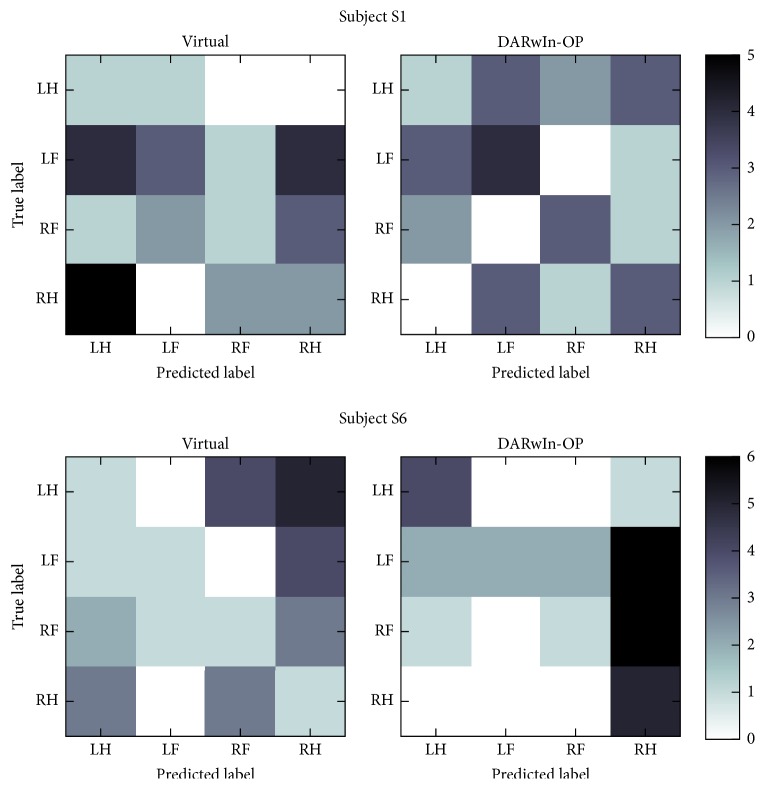
Confusion matrices for the two subjects showing the most improvement between the virtual and DARwIn-OP online BCI results. The confusion matrices indicate different strategies for improving accuracy during DARwIn-OP control: S1 shows a mostly diagonal pattern while S6 shows a focus on correct classification of the two hand tasks.

Subjects S1 and S6, who showed the largest improvement between the virtual and DARwIn-OP BCIs, have confusion matrices that indicate differing methods used to increase accuracy. The confusion matrix of online classification results for subject S1 shows a strong diagonal pattern when controlling the DARwIn-OP, as expected for a well-performing classifier. Interestingly, left foot and right foot are never misclassified as the opposite foot, as might be expected based on their close proximity in homuncular organization, even though such misclassifications were present when controlling the virtual robot. Left hand was the most frequently misclassified task, commonly confused with left foot and right hand. Left foot tasks were also misclassified as left hand tasks but were correctly classified much more often. Subject S6, on the other hand, achieved higher accuracy when controlling the DARwIn-OP by primarily classifying the two hand tasks correctly. This subject's classifier had a strong tendency to predict right hand tasks during both BCIs, although actual right hand tasks were often misclassified during virtual robot control. The two foot tasks in both scenarios were frequently misclassified, typically as right hand. The confusion matrices are shown in Figure 9.

## 4. Discussion

In this work, we present the results of a four-class motor-imagery-based BCI used to control a virtual and physical robot. There were significant differences in performance between controlling the virtual robot and the physical DARwIn-OP robot with the BCI. Subjects had significantly higher accuracy when controlling the DARwIn-OP than when controlling the virtual robot (29.72% versus 24.51% accuracy, resp.). An offline analysis showed that the interaction between optode and robot type had a significant effect on HbO levels, indicating that this increase in accuracy may be at least partially due to changes in HbO activation patterns during the tasks. Topographic plots of HbO activation also show changes in activation pattern between the virtual and DARwIn-OP BCIs, with left hand and right foot tasks moving to a more contralateral activation pattern while right hand and left foot became more ipsilateral in the second BCI.

These changes could be due to the participants adapting their mental strategy based on the BCI's classifier while controlling the virtual robot, thereby modifying their motor-imagery activation patterns. Confusion matrices of the online BCI classifiers show different patterns of correct and incorrect classification between subjects and between control of the virtual and physical robot. Such changes could reflect differences in the activation patterns generated during motor imagery, potentially showing differences in mental strategy developed by the participants while using the BCIs. This is in line with previous findings that feedback, especially from a BCI, can improve motor-imagery activation [49, 52, 64, 65]. Participants could also have improved as they became more familiar with the BCI experiment protocol, increasing their confidence in using the BCI, which has also been shown to have an effect on motor-imagery ability [45].

It is also possible that the differences between the virtual and DARwIn-OP robots themselves contributed to differences in subject performance. The more realistic visuals when using the DARwIn-OP could have had an effect, similar to the results found by Alimardani et al. [52]. There has been limited study on this topic, and further experiments would be needed in order to determine if this was a factor in subject performance.

There was a large difference between the accuracy of the highest-accuracy and lowest-accuracy subjects (40% versus 16% accuracy), in line with previous findings that people have different motor-imagery abilities [45–47]. Future studies could be improved by screening participants for motor-imagery abilities, as suggested by Marchesotti et al. [46], and potentially using feedback to improve the performance of participants identified as low motor-imagery ability [48]. As Bauer et al. found that the use of a robot BCI could improve motor-imagery performance, longer or additional BCI sessions could be incorporated in order to improve motor-imagery performance [49].

In this work, we adapted the preprocessing pipeline for each subject based on classifier performance on the two training days. While this allows one more element of customization for each subject-specific classifier, it also increases the likelihood of overfitting on the training data, which can result in poor performance on the online BCI. Future work could compare the different preprocessing methods and select a single method that performs best across subjects. Additionally, the ability to distinguish between four motor-imagery tasks with simple descriptive features and classifiers may be limited. Future work could employ more intelligent feature reduction methods (e.g., Sequential Floating Forward Selection) or explore more powerful feature design methods using deep neural networks or autoencoders. Support vector machines with nonlinear kernels may be able to achieve higher classification accuracy than LDA classifiers. The more powerful classification abilities of neural networks may also prove beneficial for improving BCI performance, as has been explored recently with EEG-based BCIs [66–69].

## 5. Conclusions

This study reports the first online results of a motor-imagery-based fNIRS-BCI to control robot navigation using four motor-imagery tasks. Subjects used the BCI to control first a virtual avatar and then a DARwIn-OP humanoid robot to navigate to goal locations within a series of three rooms. Classification accuracy was significantly greater during the DARwIn-OP BCI, and an offline analysis found a significant interaction between optode and both task and robot type on HbO activation levels. These findings corroborate previous studies that show feedback, including feedback from controlling a robot BCI, can improve motor-imagery performance. It is also possible that the use of a physical, as opposed to virtual, robot had an effect on the results, but future study would be needed to assess that. Furthermore, the activation patterns for left hand and right foot change to show a more strongly contralateral activation pattern during the second BCI, becoming more in line with the expected activation patterns based on the cortical homunculus layout of the motor cortex.

These findings indicate that future studies could benefit from additional focus on feedback during training and in particular additional training periods spent controlling the actual BCI. There was also a large discrepancy between the accuracy of the highest-accuracy and lowest-accuracy subject, indicating that future studies could be improved by screening potential subjects for BCI abilities and potentially providing these subjects with extra feedback training.

## Supplementary Material

Results tables for post-hoc analyses.

## Figures and Tables

**Table 1:** Effects and interactions of task, optode, and robot type for the BCI control session.

	*F*-value	*p* value
Task	0.011	0.998
Optode	10.982	0.000
Robot type	0.043	0.835
Optode*∗*task	1.393	0.018
Task*∗*robot type	0.008	0.999
Optode*∗*robot type	2.155	0.001
Optode*∗*task*∗*robot type	1.147	0.192

**Table 2:** Online BCI results.

	Accuracy	Precision	Recall	*F*-Score	AUC
S1	30.00	0.31	0.29	0.30	0.50
S2	27.12	0.32	0.29	0.30	0.50
S3	25.00	0.19	0.25	0.22	0.47
S4	21.67	0.30	0.26	0.28	0.50
S5	30.00	0.28	0.28	0.28	0.54
S6	26.67	0.37	0.28	0.32	0.50
S7	35.00	0.36	0.37	0.37	0.59
S8	36.67	0.34	0.32	0.33	0.53
S9	18.33	0.22	0.21	0.21	0.54
S10	20.00	0.20	0.21	0.20	0.45
S11	31.67	0.22	0.25	0.24	0.49
S12	23.33	0.23	0.23	0.23	0.52
Avg.	27.12	0.28	0.27	0.27	0.51

**Table 3:** Online BCI results for virtual and DARwIn-OP BCIs individually.

	Virtual robot					DARwIn-OP robot				
	Accuracy	Precision	Recall	*F*-Score	AUC	Accuracy	Precision	Recall	*F*-Score	AUC
S1	23.33	0.27	0.28	0.27	0.51	36.67	0.36	0.38	0.37	0.53
S2	24.14	0.24	0.23	0.23	0.40	30.00	0.44	0.34	0.38	0.63
S3	23.33	0.16	0.25	0.19	0.55	26.67	0.20	0.23	0.21	0.43
S4	26.67	0.36	0.44	0.40	0.59	16.67	0.17	0.18	0.18	0.47
S5	30.00	0.29	0.23	0.25	N/A	30.00	0.35	0.31	0.33	0.57
S6	13.33	0.21	0.14	0.17	0.42	40.00	0.55	0.52	0.53	0.65
S7	33.33	0.35	0.34	0.35	0.59	36.67	0.46	0.39	0.42	0.57
S8	33.33	0.29	0.30	0.29	0.53	40.00	0.40	0.35	0.38	0.54
S9	16.67	0.16	0.22	0.18	0.52	20.00	0.25	0.20	0.22	0.55
S10	20.00	0.17	0.22	0.20	0.42	20.00	0.20	0.17	0.18	0.48
S11	30.00	0.23	0.25	0.24	0.44	33.33	0.19	0.27	0.22	0.54
S12	20.00	0.21	0.19	0.20	0.50	26.67	0.26	0.30	0.28	0.58
Avg.	24.51	0.24	0.26	0.25	0.50	29.72	0.32	0.30	0.31	0.54
