# Supplementary material for: Virtual and Actual Humanoid Robot Control with Four-Class Motor-Imagery-Based Optical Brain-Computer Interface
Source: Biomed Res Int. 2017 Jul 18;2017:1463512. doi: 10.1155/2017/1463512 (PMC5539938; doi:10.1155/2017/1463512)
Supplement: Supplementary file 1 — Results tables for post-hoc analyses. [file 1463512.f1.pdf]

Supplementary Material

for

Virtual and Actual Humanoid Robot Control with Four-Class Motor-Imagery Based Optical Brain Computer Interface

Alyssa M. Batula, Youngmoo E. Kim, Hasan Ayaz

Contents

- **Table S1:** Post-hoc analysis of Task, Robot Type, and Task*Robot Type
- **Table S2:** Post-hoc analysis of Robot Type

- **Table S1:** Post-hoc analysis of Task, Robot Type, and Task*Robot Type. Effect of Task (4 levels: left hand, left foot, right foot, right hand), Robot Type (2 levels: virtual, DARwIn-OP) and their interaction on HbO activation for individual optodes. Significant effects (p<0.05, FDR corrected) are highlighted in bold.

	Task		Robot Type		Task*Robot Type	
**Optode**	**F-value**	**p-value**	**F-value**	**p-value**	**F-value**	**p-value**
**1**	0.9408	0.5522	0.2924	0.6941	0.4105	0.8948
**2**	0.9346	0.5522	1.0982	0.5463	0.8245	0.7127
**3**	1.7630	0.4717	1.8804	0.4580	2.4475	0.5186
**4**	0.9346	0.5522	5.9306	0.1258	0.8028	0.7127
**5**	0.9963	0.5522	0.0479	0.8812	0.2471	0.9298
**6**	2.6925	0.3333	1.2457	0.5463	0.0336	0.9917
**7**	0.7741	0.6119	0.8556	0.6107	1.4346	0.6509
**8**	2.3763	0.3400	8.7333	0.0829	1.0739	0.6931
**9**	3.0851	0.3333	0.6942	0.6223	1.6322	0.6509
**10**	0.5928	0.7089	1.1857	0.5463	1.4265	0.6509
**11**	0.9551	0.5522	2.9775	0.2942	0.2902	0.9298
**12**	0.9441	0.5522	0.2649	0.6941	0.8360	0.7127
**13**	1.2165	0.5522	0.6673	0.6223	1.5536	0.6509
**14**	0.9103	0.5522	0.0014	0.9703	1.4005	0.6509
**15**	0.1481	0.9308	0.0386	0.8812	0.6334	0.7922
**16**	1.7515	0.4717	5.3634	0.1286	4.0993	0.1764
**17**	0.1912	0.9308	3.5790	0.2393	0.2076	0.9298
**18**	3.3699	0.3333	1.5520	0.5145	1.2026	0.6924
**19**	1.1593	0.5522	0.3174	0.6941	1.0408	0.6931
**20**	2.0345	0.4397	0.4267	0.6941	2.4621	0.5186
**21**	0.9798	0.5522	7.3015	0.0894	0.4121	0.8948
**22**	0.2200	0.9308	0.4009	0.6941	1.1818	0.6924
**23**	2.5623	0.3333	2.0236	0.4580	1.8086	0.6509
**24**	1.1079	0.5522	4.0037	0.2237	0.7825	0.7127

Table S2: Post-hoc analysis of Robot Type. Effect of Robot Type (2 levels: virtual, DARwIn-OP) on HbO activation for individual optodes and tasks. Significant effects (p<0.05, FDR corrected) are highlighted in bold.

Optode	Left Hand	Left Foot	Right Foot	Right Hand
**1**	0.9807	0.6630	0.5577	0.8224
**2**	0.1448	0.1358	0.9004	0.3222
**3**	0.4626	0.5369	0.0642	0.2045
**4**	0.5086	0.4521	**0.0194**	0.4521
**5**	0.8119	0.6378	0.2193	0.9781
**6**	0.2530	0.7184	0.8019	**0.0261**
**7**	0.5958	0.3246	0.8019	0.4311
**8**	0.1304	0.7213	0.6216	0.5574
**9**	0.7780	0.4521	**0.0386**	0.5442
**10**	0.7267	0.5878	0.5574	0.9004
**11**	0.5574	0.1464	0.2451	0.3952
**12**	0.4785	0.3536	0.0532	0.5369
**13**	0.8224	0.6638	0.2451	0.5086
**14**	**0.0261**	**0.0386**	0.6216	0.5574
**15**	0.8655	0.8119	0.4961	0.8119
**16**	**0.0261**	0.6216	**0.0396**	0.1740
**17**	0.7856	0.8576	0.5369	0.6354
**18**	0.2451	0.4455	**0.0325**	0.5574
**19**	0.8119	0.4961	0.9807	0.6216
**20**	0.9334	0.1308	**0.0261**	0.9614
**21**	0.4723	0.8052	0.5087	0.7267
**22**	0.5146	0.4521	0.6216	0.5442
**23**	0.6636	0.4961	**0.0261**	0.6493
**24**	0.4740	0.5756	0.1308	0.5577
